# Neoadjuvant Chemoradiotherapy Does Not Contribute to Worse Survival in Pathological Node-Negative Rectal Cancer

**DOI:** 10.3389/fonc.2021.649313

**Published:** 2021-03-08

**Authors:** Yong Huang, Wei Wei, Zhenguang Wang, Tao Liang, Shuyun Tian, Guangshun Fu

**Affiliations:** Department of General Surgery, Jiangdu People' s Hospital Affiliated to Yangzhou University Medical School, Yangzhou, China

**Keywords:** neoadjuvant chemoradiotherapy, rectal cancer, ypN0, pN0, propensity score matching

## Abstract

**Purpose:** The prognostic significance of ypN0 rectal cancer with comparison to pN0 disease still remains poorly defined. This study aimed to compare the prognosis of ypN0 and pN0 rectal cancer.

**Methods:** Eligible patients were identified from the SEER18 registries research database (the latest data up to date was on April 15, 2019). Propensity score (PS) matching was usually performed to reduce the imbalance and potential confounding that were introduced by inherent differences between the groups. The cause-specific survival (CSS) was analyzed to evaluate the prognostic prediction of ypN0 and pN0 groups using the Kaplan–Meier method with the log-rank test. Cox proportional hazard model was also used to identify independent prognostic variables.

**Results:** In total, 26,832 patients diagnosed with pN0 or ypN0 rectal cancer were confirmed as the final cohort, including 7,237 (27.0%) patients with radiation and 19,595 (73.0%) patients without radiation prior to surgery. The median follow-up time was up to 81 months. After adjusting for other prognostic factors, neoadjuvant radiotherapy was not an independent prognostic variable of CSS (HR = 1.100, 95%CI = 0.957–1.265, *P* = 0.180, using pN0 group as the reference).

**Conclusions:** ypN0 rectal cancer was strongly associated with worse pathological diagnoses compared with pN0 rectal cancer, contributing to worse oncologic outcomes. However, the receipt of neoadjuvant chemoradiotherapy was not an independent prognostic factor of worse prognosis in pathological node-negative patients. Our study could give guidance to the treatment of ypN0 rectal cancer.

## Introduction

Colorectal cancer was one of the most frequently diagnosed malignances around the world (1, 2). Due to the different anatomical location characteristics of the rectum from colon, the treatment of rectal cancer is more complex.

Currently, neoadjuvant chemoradiotherapy followed by total mesorectal excision (TME) has been widely accepted as the standard treatment for locally advanced rectal cancer (3, 4). And the histopathological evaluation of TME resection specimens played a vital role in evaluating the prognosis of rectal cancer after neoadjuvant chemoradiotherapy, which was highly dependent on the accurate assessment of postoperative lymph node status (5).

Previous studies had shown that lymph node-negative rectal cancer after neoadjuvant chemoradiation therapy (ypN0) was associated with an excellent prognosis, and the 5-year disease-free survival ranged from 79.8 to 87% (6–8). Later in 2014, with a retrospective analysis of a total of 473 patients diagnosed with rectal cancer, Erlenbach-Wünsch et al. (9) found that ypN0 rectal cancer could achieve similar oncologic results compared with pN0 disease, which suggested that adjuvant chemotherapy for ypN0 might result in overtreatment. However, this study had just a small sample size and needs to be validated in other studies, and the prognostic significance of ypN0 rectal cancer with comparison to pN0 disease still remains poorly defined (9). Here, therefore, using the newly released large population-based cancer database, we conducted this propensity score (PS) matched study to compare the prognosis of ypN0 and pN0 rectal cancer.

## Methods

### Ethics

The present study complied with the Declaration of Helsinki. All authors reviewed and approved the final edition of this manuscript. The US Surveillance, Epidemiology, and End Results (SEER) database of the National Cancer Institute (NCI) was an open public database, and the release of data from the SEER database did not require informed patient consent because cancer was a reportable disease in every state of the USA.

### Patients

As a population-based cancer registration system, the US SEER database of the NCI provides different datasets on cancer demographic information and survival, covering approximately 28% of US populations. Using the SEER^*^ Stat 8.3.5 software, we identified patients from the SEER18 registries research database (the latest data up to date was on April 15, 2019). The SEER18 database contained data from the SEER9 registries, the SEER13 registries (SEER 9 plus Los Angeles, San Jose-Monterey, Rural Georgia, and the Alaska Native Tumor Registry), and the registries of Greater California, Kentucky, Louisiana, New Jersey, and Greater Georgia (10). Patients' characteristics including No. of LNs dissected, American Joint Committee on Cancer *T*-stage (T1, T2, T3, and T4), age at diagnosis (years), race (white, black, and other), gender (male and female), year of diagnosis (2004–2011), tumor site (rectosigmoid primary and rectal primary), grade (well/Moderate, poor/anaplastic, and unknown), chemotherapy, serum carcinoembryonic antigen (CEA) level (negative, positive, and unknown), tumor size (≤5, >5 cm, and unknown) and perineural invasion (no, yes, and unknown) were obtained from the SEER database.

**Figure 1:**
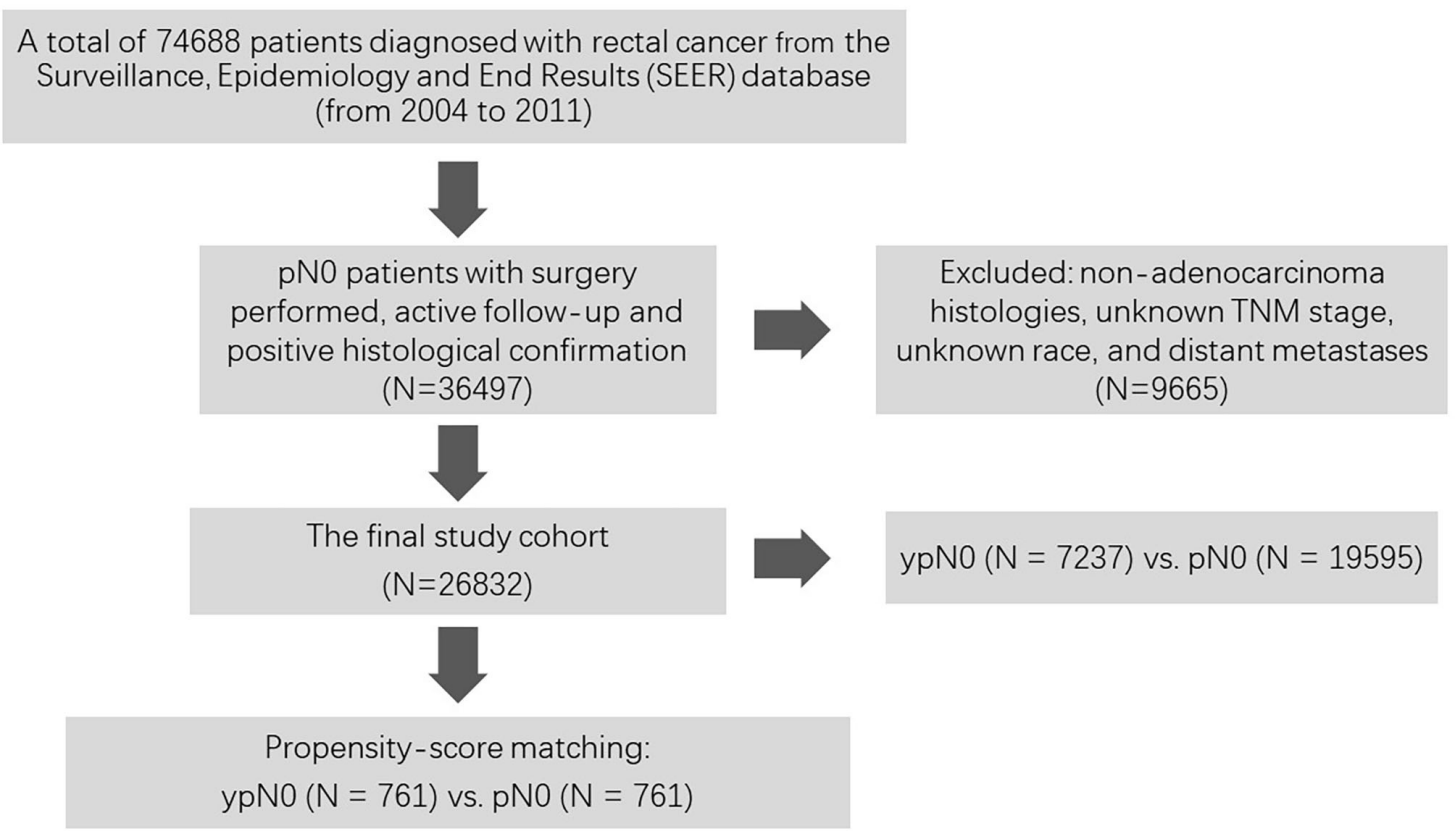
Flow diagram of the patient selection and research design.

As shown in Figure 1, at first, a total of 74,688 patients diagnosed with rectal cancer between 2004 and 2011 were identified from the Surveillance, Epidemiology, and End Results (SEER) database. Then, patients with surgery performed, active follow-up, positive histological confirmation, and pathological N0 status were included into our analyses. Those with non-adenocarcinoma histologies, unknown TNM stage, unknown race, and distant metastases were excluded from the present study. Among them, patients with (*n* = 7,237) or without (*n* = 19,595) radiation prior to surgery were confirmed as the final cohort.

### Propensity-Score Matching

**Figure 2:**
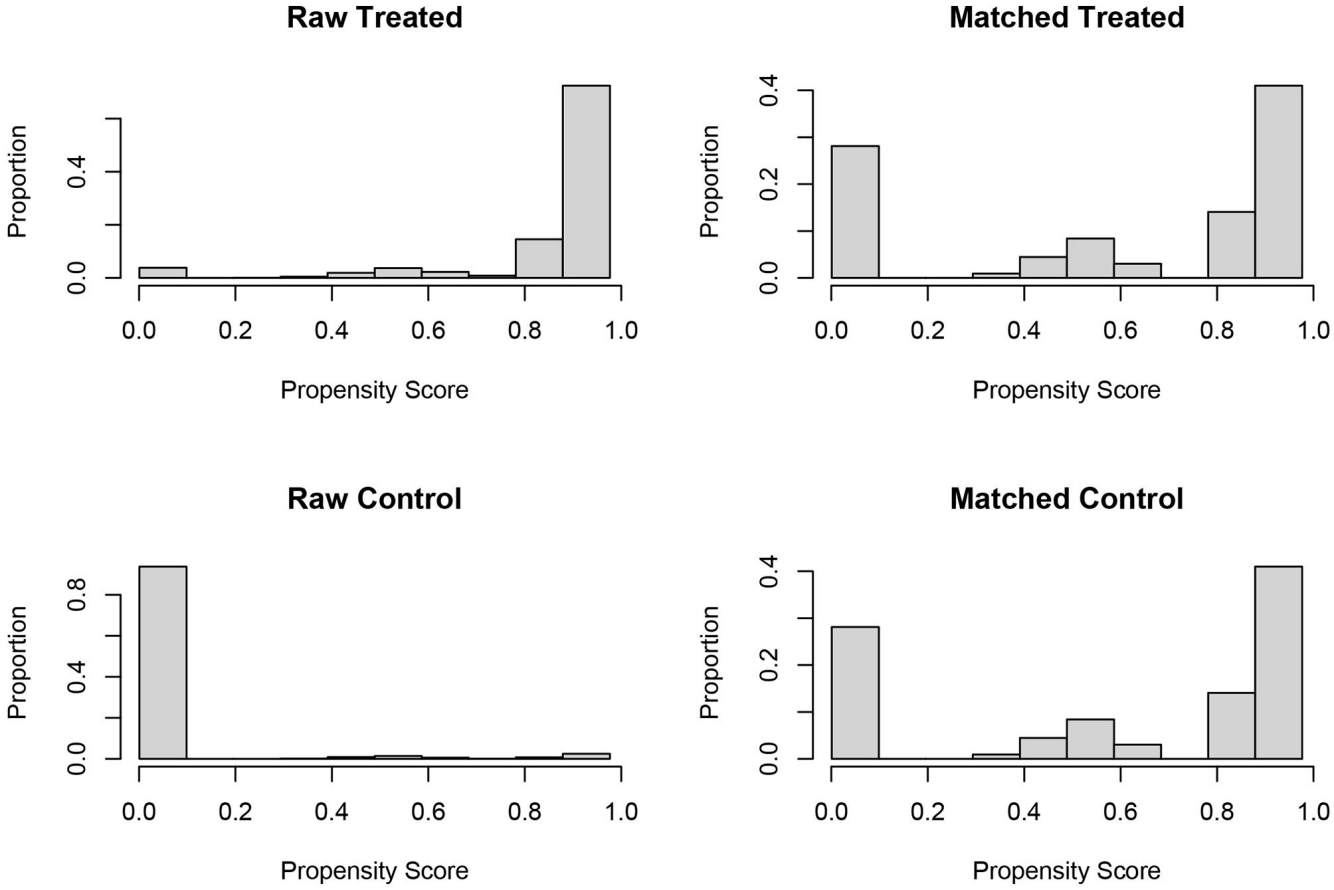
Histograms of propensity score before and after the PS matching.

In the analyses of retrospective cohort without randomization, propensity score (PS) matching was usually performed to reduce the imbalance and potential confounding that were introduced by inherent differences between the groups (11). In the present study, one to one PS matching was also used to reduce selection bias in patient characteristics between ypN0 and pN0 groups based on the following covariates: No. of LNs dissected, American Joint Committee on Cancer T stage (T1, T2, T3, and T4), age at diagnosis (years), race (white, black, and other), gender (male and female), year of diagnosis (2004–2011), tumor site (rectosigmoid primary, and rectal primary), grade (well/Moderate, poor/anaplastic, and unknown), chemotherapy, serum carcinoembryonic antigen (CEA) level (negative, positive, and unknown), tumor size (≤5, >5 cm, and unknown) and perineural invasion (no, yes, and unknown). PS matching was performed based on nearest-neighbor matching, propensity scores reflected the probability that patients would be in ypN0 and pN0 groups based on their baseline characteristics. Once the propensity scores were estimated, patients in the pN0 group were matched to patients with radiation prior to the surgery. The histograms of propensity score before and after PS matching were shown in Figure 2. Finally, 761 matched pairs (761 patients in ypN0 group and 761 patients in pN0 group) were selected from the whole cohort (*n* = 26,832).

### Statistical Analyses

The differences in the baseline characteristics between the ypN0 and pN0 groups were analyzed using the Pearson's chi-square test. The causes of death in the present study were categorized as rectal cancer specific or non–rectal cancer related. Rectal cancer cause-specific survival (CSS) was calculated from the date of diagnosis to the date of death due to rectal cancer. However, patients who died of other causes were censored at the date of death.

In our analyses, the CSS was analyzed to evaluate the prognostic prediction of ypN0 and pN0 groups using the Kaplan–Meier method with the log-rank test. The prognostic variables were entered in the multivariable analyses using the Cox proportional hazard model to identify independent prognostic variables. All the hazard ratios (HRs) were shown with 95% confidence intervals (CI). All tests were two sided, and two-sided *P*-values <0.05 were considered to be statistically significant in our analyses. Statistical analyses were mainly performed using SPSS version 23 (IBM, Armonk, NY, USA).

## Results

### Patient Characteristics Before PS Matching

In total, 26,832 patients diagnosed with pN0 or ypN0 rectal cancer were confirmed as the final cohort, including 7,237 (27.0%) patients with radiation and 19,595 (73.0%) patients without radiation prior to surgery. 8,177 (30.5%) patients received chemotherapy and 18,655 (69.5%) patients did not. The median follow-up time was up to 81 months, which was more than 5 years. At the end of follow-up time, 3,453 (12.9%) patients died of rectal cancer. The 5-year CSS rate of the whole cohort was 89.8%. The median ages of ypN0 group and pN0 group were 68 and 62 years old, respectively.

Shown as Table 1, patient demographics and pathological features between ypN0 and pN0 groups were summarized. For the number of lymph nodes dissected in total, patients in the ypN0 group were more likely to be associated with <12 lymph nodes dissected than patients in the pN0 group (*P* = 0.014); for American Joint Committee on Cancer (AJCC) *T*-stage, patients in the ypN0 group were more likely to be associated with higher *T*-stage than patients in the pN0 group (*P* < 0.001); for postoperative tumor grade, patients in the ypN0 group were more likely to be associated with higher postoperative tumor grade than patients in the pN0 group (*P* < 0.001). The above findings showed that ypN0 was strongly associated with advanced postoperative clinicopathological characteristics.

**Table 1:** Patients' baseline characteristics before PSM.

**Variables**	**No. of Patients (%)**		***P***
	**pN0 (19,595)**	**ypN0 (7,237)**	
No. of LNs dissected			0.014
<12	10,961 (55.9)	4,169 (57.6)	
≥12	8,634 (44.1)	3,068 (42.4)	
*T*-stage			<0.001
T1	8,588 (43.8)	936 (12.9)	
T2	5,351 (27.3)	1,439 (19.9)	
T3	5,085 (26.0)	4,374 (60.4)	
T4	571 (2.9)	488 (6.7)	
Age at diagnosis (years)			<0.001
≤ 65	8,490 (43.3)	4,366 (60.3)	
>65	11,105 (56.7)	2,871 (39.7)	
Race			0.018
White	16,390 (83.6)	5,951 (82.2)	
Black	1,521 (7.8)	596 (8.2)	
Other	1,684 (8.6)	690 (9.5)	
Gender			<0.001
Male	10,872 (55.5)	4,555 (62.9)	
Female	8,723 (44.5)	2,682 (37.1)	
Year of diagnosis			<0.001
2004	2,620 (13.4)	703 (9.7)	
2005	2,598 (13.3)	766 (10.6)	
2006	2,481 (12.7)	877 (12.1)	
2007	2,480 (12.7)	987 (13.6)	
2008	2,490 (12.7)	920 (12.7)	
2009	2,358 (12.0)	1,022 (14.1)	
2010	2,349 (12.0)	1,051 (14.5)	
2011	2,219 (11.3)	911 (12.6)	
Tumor site			<0.001
Rectosigmoid primary	7,483 (38.2)	691 (9.5)	
Rectal primary	12,112 (61.8)	6,546 (90.5)	
Grade			<0.001
Well/moderate	16,300 (83.2)	5,675 (78.4)	
Poor/anaplastic	1,656 (8.5)	733 (10.1)	
Unknown	1,639 (8.4)	829 (11.5)	
Chemotherapy			<0.001
No/unknown	18,377 (93.8)	278 (3.8)	
Yes	1,218 (6.2)	6,959 (96.2)	
Serum CEA level			<0.001
Negative	6,566 (33.5)	2,631 (36.4)	
Positive	2,437 (12.4)	1,744 (24.1)	
Unknown	10,592 (54.1)	2,862 (39.5)	
Tumor size			<0.001
≤ 5 cm	12,156 (62.0)	4,141 (57.2)	
>5 cm	2,701 (13.8)	1,120 (15.5)	
Unknown	4,738 (24.2)	1,976 (27.3)	
Perineural invasion			<0.001
No	3,598 (18.4)	1,508 (20.8)	
Yes	129 (0.7)	91 (1.3)	
Unknown	15,868 (81.0)	5,638 (77.9)	

In addition, postoperative lymph node negative patients who were aged <65 years old, black, male, diagnosed in later years, rectal primary and received chemotherapy correlated with higher probability to have received neoadjuvant treatment.

### Prognosis of ypN0 and pN0 Groups Before PS Matching

**Figure 3:**
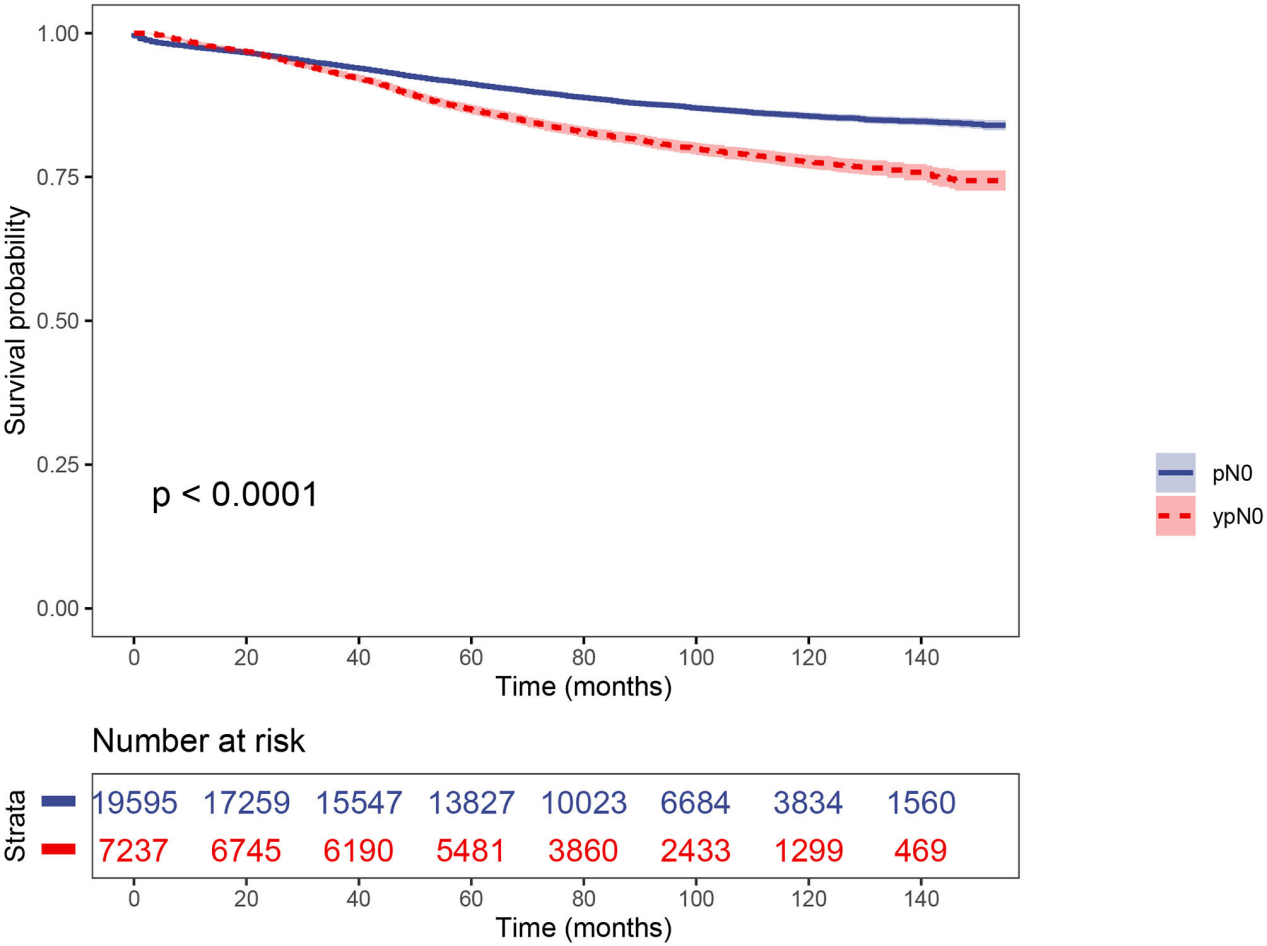
The CSS curves of ypN0 and pN0 groups using Kaplan-meier estimates before PSM.

Using Kaplan–Meier estimates, we analyzed the CSS between ypN0 and pN0 groups. Patients in the ypN0 group had significantly worse survival compared with patients in the pN0 group: the 5-year CSS rate of ypN0 and pN0 were 86.6 and 91.1%, respectively, (*P* < 0.001, Figure 3). Then, results of multivariable analyses using the Cox proportional hazard were summarized in Table 2. No. of LNs dissected <12 (HR =0.700, 95%CI = 0.650–0.753, *P* < 0.001 for No. of LNs dissected ≥ 12, using No. of LNs dissected <12 as the reference), higher *T*-stage (HR = 1.518, 95%CI = 1.359–1.695, *P* < 0.001 for T2 stage; HR = 2.439, 95%CI = 2.194–2.712, *P* < 0.001 for T3 stage; HR = 5.353, 95%CI = 4.619–6.204, *P* < 0.001 for T4 stage; using T1 stage as the reference), aged than 65 years old (HR = 1.697, 95%CI = 1.582–1.820 for age at diagnosis > 65, *P* < 0.001, using age at diagnosis ≤ 65 as the reference), black (HR = 1.457, 95%CI = 1.305–1.626, *P* < 0.001 for black race, using white race as the reference), rectal primary (HR = 1.159, 95%CI = 1.068–1.257, *P* < 0.001 for rectal primary, using rectosigmoid primary as the reference), and higher tumor grade (HR = 1.360, 95%CI = 1.228–1.506, *P* < 0.001 for poor/anaplastic grade, using well/moderate grade as the reference) were independently associated with significantly worse CSS. With regards to neoadjuvant radiotherapy, however, after adjusting for other prognostic factors, it was not an independent prognostic variable of CSS (HR = 1.095, 95%CI = 0.952–1.260, *P* = 0.205, using pN0 group as the reference).

**Table 2:** Multivariate Cox regression analyses of the clinicopathological characteristics concerning CSS.

**Variable**	**Reference**	**Characteristic**	**Cause-specific survival**		
			**HR (95%CI)**	**SE**	***P*-value**
Neoadjuvant radiotherapy					0.205
	No	Yes	1.095 (0.952–1.260)	0.072	
No. of LNs dissected					<0.001
	<12	≥12	0.700 (0.650–0.753)	0.037	
T stage					<0.001
	T1	T2	1.518 (1.359–1.695)	0.056	<0.001
		T3	2.439 (2.194–2.712)	0.054	<0.001
		T4	5.353 (4.619–6.204)	0.075	<0.001
Age at diagnosis (years)					<0.001
	≤ 65	>65	1.697 (1.582–1.820)	0.036	
Race					<0.001
	White	Black	1.457 (1.305–1.626)	0.056	<0.001
		Other	0.884 (0.782–1.000)	0.063	0.051
Gender					
	Male	Female	0.956 (0.893–1.023)	0.035	0.195
Year of diagnosis					0.706
	2004	2005	1.083 (0.957–1.225)	0.063	0.205
		2006	1.017 (0.896–1.155)	0.065	0.791
		2007	1.042 (0.918–1.184)	0.065	0.523
		2008	1.009 (0.884–1.152)	0.068	0.897
		2009	0.994 (0.868–1.139)	0.069	0.931
		2010	1.035 (0.842–1.271)	0.105	0.746
		2011	0.923 (0.736–1.157)	0.116	0.486
Tumor site					<0.001
	Rectosigmoid primary	Rectal primary	1.159 (1.068–1.257)	0.042	
Grade					<0.001
	Well/moderate	Poor/Anaplastic	1.360 (1.228–1.506)	0.052	<0.001
		Unknown	0.859 (0.751–0.982)	0.069	0.027
Chemotherapy					0.736
	No/unknown	Yes	0.977 (0.852–1.120)	0.070	
Serum CEA level					<0.001
	Negative	Positive	1.646 (1.498–1.808)	0.048	<0.001
		Unknown	1.225 (1.131–1.327)	0.041	<0.001
Tumor size					<0.001
	≤ 5 cm	>5 cm	1.274 (1.163–1.395)	0.046	<0.001
		Unknown	1.123 (1.027–1.228)	0.045	0.011
Perineural invasion					0.008
	No	Yes	1.648 (1.205–2.254)	0.160	0.002
		Unknown	1.037 (0.849–1.265)	0.102	0.723

### Patient Characteristics and Prognosis of ypN0 and pN0 Groups After PS Matching

**Figure 4:**
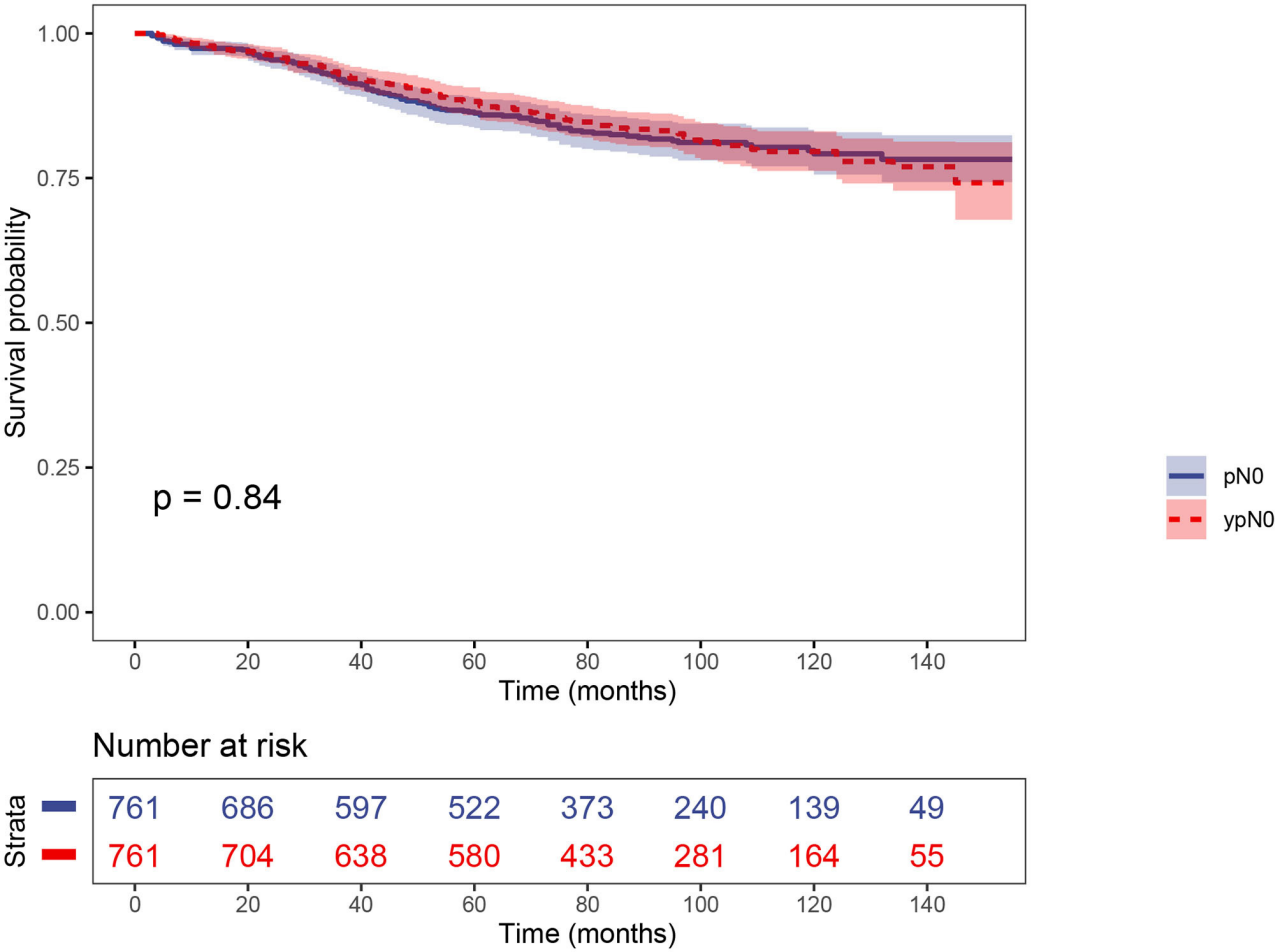
The CSS curves of ypN0 and pN0 groups using Kaplan-meier estimates after PSM.

PS matching created 761 matched pairs, including 761 patients in the ypN0 group and 761 patients in the pN0 group. The comparison of baseline characteristics between the two groups were summarized in Table 3. All the tumor and patient characteristics except year of diagnosis showed no statistically significant differences between ypN0 and pN0 groups (*P* > 0.05, Table 3). Then, we also conducted CSS analyses using the Kaplan–Meier method, which indicated that there was no statistically significant CSS difference between the two groups, the 5-year CSS rates of the ypN0 and pN0 groups were 88.2 and 86.2%, respectively, (*P* = 0.84; Figure 4).

**Table 3:** Patients' baseline characteristics after PSM.

**Variables**	**No. of Patients (%)**		***P***
	**pN0 (761)**	**ypN0 (761)**	
No. of LNs dissected			1.000
<12	395 (51.9)	395 (51.9)	
≥12	366 (48.1)	366 (48.1)	
T stage			0.888
T1	121 (15.9)	112 (14.7)	
T2	148 (19.4)	157 (20.6)	
T3	469 (61.6)	470 (61.8)	
T4	23 (3.0)	22 (2.9)	
Age at diagnosis (years)			0.756
≤65	428 (56.2)	434 (57.0)	
>65	333 (43.8)	327 (43.0)	
Race			0.864
White	667 (87.6)	671 (88.2)	
Black	45 (5.9)	46 (6.0)	
Other	49 (6.4)	44 (5.8)	
Gender			0.316
Male	479 (62.9)	460 (60.4)	
Female	282 (37.1)	301 (761)	
Year of diagnosis			0.944
2004	81 (10.6)	95 (12.5)	
2005	97 (12.7)	96 (12.6)	
2006	99 (13.0)	106 (13.9)	
2007	107 (14.1)	101 (13.3)	
2008	104 (13.7)	102 (13.4)	
2009	98 (12.9)	101 (13.3)	
2010	91 (12.0)	83 (10.9)	
2011	84 (11.0)	77 (10.1)	
Tumor site			0.950
Rectosigmoid primary	158 (20.8)	159 (20.9)	
Rectal primary	603 (79.2)	602 (79.1)	
Grade			0.221
Well/moderate	686 (90.1)	666 (87.5)	
Poor/anaplastic	50 (6.6)	59 (7.8)	
Unknown	25 (3.3)	36 (4.7)	
Chemotherapy			1.000
No/unknown	214 (28.1)	214 (28.1)	
Yes	547 (71.9)	547 (71.9)	
Serum CEA level			0.890
Negative	276 (36.3)	267 (35.1)	
Positive	153 (20.1)	156 (20.5)	
Unknown	332 (43.6)	338 (44.4)	
Tumor size			0.721
≤5 cm	516 (67.8)	511 (67.1)	
>5 cm	125 (16.4)	119 (15.6)	
Unknown	120 (15.8)	131 (17.2)	
Perineural invasion			0.738
No	153 (21.1)	141 (18.5)	
Yes	8 (1.1)	8 (1.1)	
Unknown	600 (78.8)	612 (80.4)	

## Discussion

The use of neoadjuvant chemoradiotherapy in advanced rectal cancer could result in pathologic response of the primary tumor, and many studies demonstrated that tumor response of neoadjuvant treatment was significantly associated with the prognosis of rectal cancer (12–16). According to the clinical guidelines of National Comprehensive Cancer Network (NCCN), patients who had received neoadjuvant chemoradiotherapy followed by surgery were recommended to receive adjuvant chemotherapy (17). However, the use of adjuvant chemotherapy in ypN0 rectal cancer was still controversial and some researchers questioned the clinical value of adjuvant chemotherapy in ypN0 patients (6, 7, 17). As early as in 2006, the study Fietkau et al. reported that disease-free survival (36 months) for rectal cancer without lymph node metastases (ypN0) was excellent, independent of whether they had received postoperative chemotherapy (6). Then in 2010, after identifying randomized studies exploring adjuvant chemotherapy against observation in patients with rectal cancer previously treated with preoperative radio(chemo)therapy, Bujko et al. (18). concluded that delivery of adjuvant chemotherapy in patients undergoing preoperative radio(chemo)therapy was not evidence based. Later, after comparing the prognosis of ypN0 patients who had received adjuvant chemotherapy and those who had not, Kiran et al. (7) found that ypN0 rectal cancer, whether or not the patient had received adjuvant chemotherapy, showed similar local recurrence, disease-free survival, and overall survival after prolonged follow-up. The famous EORTC 22921 trial's long-term results also showed that adjuvant fluorouracil-based chemotherapy after preoperative radiotherapy (with or without chemotherapy) does not affect either 10-year overall survival or disease-free survival of rectal cancer (19). Therefore, it was quite necessary to examine the long-term oncologic results of ypN0 disease.

To the best of our knowledge, the present population-based study was the largest study to compare the oncologic outcomes of ypN0 and pN0 rectal cancer. In the present study, at first, shown as the results of Kaplan–Meier estimates, patients in the ypN0 group had significantly worse survival compared with patients in the pN0 group. However, after adjusting for other known prognostic factors, the results of multivariate analyses showed that the prognostic difference between ypN0 and pN0 groups was not statistically significant. More importantly, PS matching was also used to validate our results and we found that there was no statistically significant CSS difference between the two groups after PS matching. Therefore, we held the belief that ypN0 status could achieve similarly good oncologic outcomes compared with pN0 disease. Therefore, we strongly believed that having received neoadjuvant chemoradiotherapy should not be the reason for adjuvant chemotherapy in pathological node-negative patients.

Although the nature of the retrospective design and small sample size were considered to be potential limitations, two previous studies questioned the routine use of adjuvant chemotherapy for ypN0 rectal cancer patients who had undergone curative surgery following neoadjuvant chemoradiation (6, 7). What is more, a recent analysis of the SEER database found that rectal cancer patients with ypTis-2N0 did not benefit from adjuvant chemotherapy after neoadjuvant treatment followed by radical surgery (20). Therefore, our research could add new evidence supporting the above findings.

Why did the Kaplan–Meier survival analyses, before adjusting for other prognostic variables or PS matching, show worse survival of ypN0 disease? In our analyses of differences in the baseline characteristics between the ypN0 and pN0 groups, we could easily find that, compared with pN0 rectal cancer, ypN0 status was strongly associated with poorer postoperative pathological diagnoses: ypN0 was more likely to be associated with <12 lymph nodes dissected, higher T stage and higher postoperative tumor grade. Before adjusting for other prognostic factors, therefore, it was normal to find that ypN0 disease was more likely to be associated with worse oncologic outcomes compared with pN0 rectal cancer.

In 2014, Erlenbach-Wünsch et al. (9) retrospectively analyzed the prognosis of 132 rectal cancer patients who underwent standard TME surgery after neoadjuvant chemoradiotherapy (ypN0) and those of 341 patients diagnosed with pN0 rectal disease without neoadjuvant chemoradiotherapy, showing a similar oncologic outcome between the two groups, which was consistent with our analyses. However, the sample size of this study was still too small for any general recommendation. Maybe limited to the sample size, they did not find that ypN0 status was strongly associated with poorer postoperative pathological diagnoses (less lymph nodes dissected, higher T stage and higher postoperative tumor grade) compared with pN0 rectal cancer, which contributed to the phenomenon that ypN0 disease was more likely to be associated with worse oncologic outcomes than pN0 rectal cancer before adjusting for other prognostic factors.

Although previous research had showed that the histological lymph node status after chemoradiotherapy seemed to be the only significant prognostic parameter of oncologic outcomes, to our knowledge, few studies were reported to study on the prognostic value of ypN0 status (6). In 2007, with the analyses of 35 patients who underwent neoadjuvant chemoradiotherapy followed by excisional surgery with TEM for rectal cancer, Caricato et al. (21) reported the effect of preoperative chemoradiotherapy on postoperative lymph node status, though the prognostic assessment was not performed due to the low case number.

Lindebjerg et al. (22) reported that rectal cancer patients with a major tumor response and no lymph node metastases after treatment had a survival rate of 100% compared to 60% in the group of patients with major response but lymph node metastases after surgery. Like ypCR patients, ypN0 patients were reported to achieve significantly better oncologic outcomes compared with lymph node-positive patients (17). Sprenger et al. (23) shared the similar view that residual nodal status was the most important predictor of individual outcome after analyzing the effect of preoperative and pathological nodal status on disease-free and overall survival in 496 patients with rectal adenocarcinoma identified from a prospective database.

Our research, therefore, as the largest one focused on the comparison of prognosis between ypN0 and pN0 rectal cancer, could add strong evidence that the receipt of neoadjuvant chemoradiotherapy was not an independent prognostic factor in rectal cancer patients with negative pathological nodal status. However, ypN0 status was strongly associated with worse postoperative pathological diagnoses compared with pN0 rectal cancer: ypN0 was more likely to be associated with <12 lymph nodes dissected, higher T stage higher postoperative tumor grade, contributing to the phenomenon that ypN0 disease was more likely to be associated with worse oncologic outcomes than pN0 rectal cancer before adjusting for other prognostic factors.

However, this study was only a retrospective one, we hope further randomized prospective study could be conducted to provide higher grade evidence to guide the treatment of rectal cancer with negative pathological nodal status who had received neoadjuvant chemoradiotherapy followed by total mesorectal excision (TME). Moreover, regimens used in the present study were not available in SEER database, which was also a limitation of our research.

In summary, our study showed that ypN0 rectal cancer was strongly associated with worse postoperative pathological diagnoses compared with pN0 rectal cancer, contributing to worse oncologic outcomes. After adjusting for other known prognostic factors, however, the prognostic difference between ypN0 and pN0 groups was not statistically significant, which could give guidance to the treatment of ypN0 rectal cancer.

## Data Availability Statement

Publicly available datasets were analyzed in this study. This data can be found here: seer.cancer.gov.

## Author Contributions

YH and GF: conceptualization. YH and WW: data curation and writing—original draft. YH, WW, and ZW: data analysis. TL and ST: visualization. GF: writing—review and editing. All authors contributed to the article and approved the submitted version.

## Conflict of Interest

The authors declare that the research was conducted in the absence of any commercial or financial relationships that could be construed as a potential conflict of interest.
